# Deciphering the role of host species for two *Mycobacterium bovis* genotypes from the European 3 clonal complex circulation within a cattle‐badger‐wild boar multihost system

**DOI:** 10.1002/mbo3.1331

**Published:** 2022-12-15

**Authors:** Laetitia Canini, Gabriela Modenesi, Aurélie Courcoul, Maria‐Laura Boschiroli, Benoit Durand, Lorraine Michelet

**Affiliations:** ^1^ Epidemiology Unit, Laboratory for Animal Health, Anses Paris‐Est University Maisons‐Alfort France; ^2^ Tuberculosis National Reference Laboratory, Bacterial Zoonosis unit, Laboratory for Animal Health, Anses Paris‐Est University Maisons‐Alfort France; ^3^ Present address: Santé publique France, French National Public Health Agency Regional Unit (Cire) Ile‐de‐France France; ^4^ Present address: Oniris Nantes 44300 France

**Keywords:** bovine tuberculosis, multi‐host system, *Mycobacterium bovis*, phylodynamics

## Abstract

Bovine tuberculosis is a common disease affecting cattle and wildlife worldwide. *Mycobacterium bovis* circulation in wildlife decreases the efficacy of surveillance and control programs in cattle. Strains of the European 3 clonal complex are the most frequent in France. The aim of our work was hence to investigate the role played by cattle and wildlife species in the circulation of two *M. bovis* European 3 strains circulation. WGS of *M. bovis* strains collected between 2010 and 2017 in two distinct areas (Nouvelle‐Aquitaine region, NAq, and Côte‐d'Or département, CdO), from badgers, wild boars, and cattle were used in an evolutionary model to infer the transition between the three species. We computed host species transition and persistence between two consecutive nodes and the average number of transitions per tree. In total, 144 and 218 samples were collected respectively in CdO and NAq. In CdO, three between‐species transition rates stood out: from cattle to badgers, from badgers to wild boars, and from wild boars to cattle. In NAq an additional fourth transition rate was identified: from badgers to cattle. However, host transition remained a rare event. Our results suggest that wild boars could be an intermediary host between badgers and cattle in the circulation of the studied strains in CdO and NAq. Our results also highlight the differences between these two areas, suggesting that the transition pattern does not only depend on the host species and other ecological, landscape and anthropic factors are important.

## Graphical abstract

Using whole genome sequencing of *Mycobacterium bovis* strains collected in two distinct areas of France with an evolutionary model, we showed that the role of host species in the circulation of the pathogen differed between both areas. While wild boars appeared to play the role of an intermediary between badgers and cattle in both areas, the role of badgers differed. Our results suggest that the transition pattern depends on ecological, landscape, and anthropic factors.

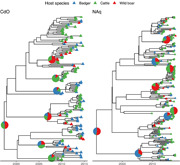

## 1 INTRODUCTION

Bovine tuberculosis (bTB) is a disease affecting cattle and wildlife worldwide (Bovine tuberculosis (2021)). *Mycobacterium bovis* can infect a large variety of wildlife hosts (Fitzgerald & Kaneene, 2013), which differ from country to country. *M. bovis* was detected in European badgers (*Meles meles*) in the UK (Rivière et al., 2014), Ireland, and continental Europe, in wild boars (*Sus scrofa*) in continental Europe (Rivière et al., 2014), in red foxes (*Vulpes vulpes*) in France (Michelet et al., 2018), in cervids, more specifically red deer (*Cervus elaphus*) and roe deer (*Capreolus capreolus*) in continental Europe (Rivière et al., 2014), Sika deer (*Cervus nippon*) recently identified in Ireland (Kelly et al., 2021) and white‐tailed deer (*Odocoileus virginianus*) and elk (*Cervus canadensis*) in North America or brush‐tailed possums (*Trichosurus vulpecula*) in New Zealand (Fitzgerald & Kaneene, 2013). The circulation of *M. bovis* in wildlife hampers control programs when implemented.

In France, a control program in line with the European Union (EU) Directive 64/432/EEC has been implemented starting in 1954 to eradicate bTB in cattle farms. This program led to a rapid decrease in herd incidence (Michelet et al., 2020), resulting in a disease‐free status in 2001 when herd prevalence was below 0.1% for six consecutive years (Decision 2001/26/EC). The bTB‐free status alleviates the control measures for export and therefore ensures French cattle farming competitiveness.

There are two surveillance programs in France: one for cattle and one for wildlife. The cattle surveillance program relies on three components. First, at slaughterhouses at the national level, all carcasses are systematically inspected, with incisions of specific tissues (lungs, retropharyngeal, tracheobronchial, and mediastinal lymph nodes). Samples from suspect lesions are sent for bTB confirmation by polymerase chain reaction (PCR) or bacteriology to certified laboratories. Second, depending on the epidemiological situation in the *département* (intermediate administrative divisions of France), periodic systematic screening of all animals over 6 weeks of age is performed at a regularity ranging from yearly to none. Finally, based on the epidemiological investigation of bTB‐infected farms, targeted screening is performed on animals before they depart from at‐risk farms (Delavenne et al., 2020a).

The wildlife surveillance program created in 2011, called Sylvatub, focuses on red deer, roe deer, wild boars, and badgers (Rivière et al., 2014). It is based on event‐based surveillance (*e.g*. carcass inspection of hunted wild boars and cervids), enhanced event‐based surveillance (e.g., carcass inspection of badgers found dead on the roadside), and programmed surveillance (e.g. badger trapping or hunted wild boar direct diagnosis). The implementation of these modalities depends on the level of surveillance defined at the *département* level, which in turn depends on their epidemiological situation. Animals are necropsied and appropriate samples are taken for bTB diagnosis by PCR and culture and molecular typing when confirmed positive (Réveillaud et al., 2018).

In France, the epidemiological situation is heterogeneous. Few areas concentrate most national outbreaks, such as Côte‐d'Or *département* in the East of France or the Nouvelle‐Aquitaine region in the South‐West. As a result, in these two areas, surveillance of cattle was biennial until 2018 and is annual since 2018 in the Dordogne *département*; and for wildlife, the program includes event‐based, enhanced event‐based, and programmed surveillance (Delavenne et al., 2020b).

In addition, molecular typing revealed that *M. bovis* strains circulating in these two regions are specific per area. For instance, SB0120 VNTR profile 5 3 5 3 9 4 5 6 (SB0120‐NAq) is mainly found in an area of Nouvelle‐Aquitaine overlapping Dordogne, Haute‐Vienne, Charente and Charente‐Maritime *départements*, while SB0120 VNTR profile 5 5 4 3 11 4 5 6 (SB0120‐CdO) is mainly found in Burgundy, especially Côte‐d'Or *département (*Michelet et al., 2020 *)*. To differentiate strains of these dominant genotypes within these regions at a finer scale, a more discriminating method is required such as whole genome sequencing (WGS), which has shown higher resolution (Crispell et al., 2017; Price‐Carter et al., 2018).

Despite being phylogenetically close genotypes belonging to the European 3 (Eu3) clonal complex (also described as lineage La1.2 (Zwyer et al., 2021)) affecting the same host species (Hauer et al., 2015, 2019), the epidemiologic situations were contrasted in these two areas. While the number of cattle outbreaks has been steadily decreasing in CdO, the number of detected cases has been increasing in NAq (Delavenne et al., 2020a). In wildlife, a similar trend was observed in badgers, with the apparent prevalence decreasing from 8.1% to 4.2% in Côte‐d'Or between 2013 and 2014 (as identified by culture) and 2016–2017 (as identified by PCR) while it increased in Nouvelle‐Aquitaine from 2.7% to 5.3% (Réveillaud et al., 2018). However, the apparent prevalence of wild boars decreased in both areas (from 3.1% to 2.2% in Côte‐d'Or and from 4.1% to 2.7% in Nouvelle‐Aquitaine during the same time intervals) (Réveillaud et al., 2018).

The drivers of these epidemic dynamics are still unclear. In particular, the role played by each species in these two multi‐host systems remains to be determined. Previous studies on bTB have focused on interactions between two species: primarily cattle and badgers (Biek et al., 2012; Bouchez‐Zacria et al., 2018; Crispell et al., 2019; Rossi et al., 2020; Trewby et al., 2014) but also cattle and possums (Crispell et al., 2017), cattle and elk (Salvador et al., 2019), cattle and cervids (Crispell et al., 2020), or wild boars and cervids (Zanella et al., 2008). However, species such as badgers and wild boars have different life traits: while badgers are sedentary and have a life expectancy of about 14 years, wild boars can travel long distances and are often hunted before they are 4 or 5 years old (Byrne et al., 2014; Podgórski et al., 2013). In addition, the amount and the timing of *M. bovis* shedding by the different species, hence their infectiousness, may differ. Thus, the roles played by different species in the multi‐host system could be different.

The aim of our work was therefore to investigate the role played by each species in the circulation of two Eu3 *M. bovis* lineages using genomic data. To do so, we defined two study areas in CdO and NAq, in which samples collected from cattle, badgers, and wild boars have been analyzed by WGS. We then used these data to model the evolutionary history of the pathogen and to infer host species of ancestors. This allowed us to analyze the transitions between species in a three‐species multi‐host system.

## 2 MATERIAL AND METHODS

### 2.1 bTB detection

All bTB detections which have been declared to the Directorate General on Food Safety (*Direction Générale de l'Alimentation – DGAl*) between 2010 and 2017 included herd identification for cattle and date of bTB confirmation. The National Reference Laboratory (NRL) (Anses, Maisons‐Alfort) verified the data set for consistency with the samples that are analyzed at the lab for bTB confirmation.

Suspect cattle had been identified either by skin tests (in the cervical region, using single intradermal tuberculin test (SITT) or single intradermal comparative tuberculin test (SICTT)) provided by different surveillance protocols (periodic screening, epidemiological investigations, pre‐movement of cattle (Delavenne et al., 2020a)) or following the detection of lesions at the slaughterhouse. For each detection, the presence of *M. bovis* was confirmed by PCR and/or bacterial culture (Delavenne et al., 2020a). Isolated strains are genotyped by spoligotyping and MIRU‐VNTR. Only isolates belonging to the SB0120 spoligotype were included, more specifically isolates of genotype SB0120‐CdO in CdO and isolates of genotype SB0120‐NAq in NAq.

Wildlife animals shot during hunting or found dead were subject to a necropsy and samples were collected to detect mycobacteria by PCR and culture. All strains isolated from badgers and wild boars identified during the wildlife surveillance program in the study area were included in our study. We decided to exclude samples collected from red deer, roe deer, and red foxes since their limited number (*n* = 2, 1, and 5, respectively) would have altered parameter inference (Réveillaud, 2013).

### 2.2 Study areas selection

In CdO and NAq, study areas were defined according to the following criteria: (1) the municipality with the most isolates was included; (2) the final study area was well delimited (i.e., it was mostly surrounded by municipalities without detected cases); (3) the final study area was compact (i.e., with a limited number of municipalities without cases detected within it). The municipalities with detected cases were municipalities where infected wildlife was identified or municipalities with pastures belonging to farms with outbreaks. The pastures were identified with the 2013–2018 French graphic parcel register (Relevé Parcellaire Graphique, RPG) provided by the French Ministry of Agriculture. We call below “outbreak” the official declaration of one or several bTB‐infected animals in a given farm. A farm could hence have several outbreaks (in case of breakdown after the farm has recovered the bTB‐free status). We limited the number of samples to be analyzed per outbreak to three. Indeed, in NAq, the number of available isolates per outbreak varied between one and 26 and was <3 in most outbreaks (84%). As two or three different VNTR profiles were identified in some outbreaks (2.5%), bounding the number of isolates per outbreak to three allowed limiting the number of isolates to sequences, while allowing for the detection of different sequences in the same outbreak.

**Figure 1:**
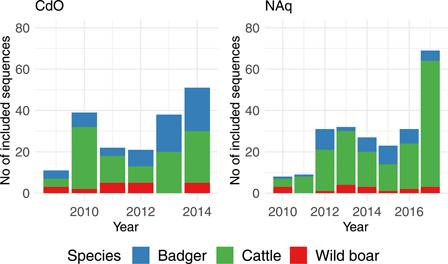
Temporal distribution of sequences for each species (blue for badgers, green for cattle, and red for wild boars) included in the study areas of Côte‐d'Or (left) and Nouvelle‐Aquitaine (right).

CdO was the pilot area and the municipalities were chosen manually according to the detection of infected wildlife or localization of pastures belonging to infected farms. In CdO, the selected area consisted of 38 municipalities, covering 499 km², in which 144 isolates (only one *M. bovis* strain per individual animal) were selected between 2009 and 2014. The number of samples to be analyzed was limited to three per outbreak. The host species were cattle (*n* = 77, from 74 outbreaks), badgers (*n* = 52) and wild boars (*n* = 15) (Figure 1, left panel) (Table A1).

For NAq, we decided to formalize the municipality selection process with a procedure that reflects the decisions taken to select the municipalities in CdO. First, for each municipality, the number of samples available was defined as the total of available samples collected from the outbreak and the wildlife. We then defined the “starting zone” as the municipality with the most available samples and the neighboring municipalities. We iteratively added one municipality randomly selected from the list of neighboring municipalities with at least one sample. The added municipality was thus adjacent to the previously defined area. Iterations stopped once a predefined maximal number of samples to include (*n*_max_) was reached. We ran this iterative process 1000 times. Among the 1000 areas thus obtained, we selected the best‐delineated area, minimizing the ratio of the total length of borders with noninfected municipalities to the perimeter of the selected area.

The resulting selected area in NAq consisted of 76 municipalities, covering 1540 km², in which *n*_max_ = 219 samples collected between 2010 and 2017 were selected. The host species were cattle (*n* = 161, from 95 outbreaks), badgers (*n* = 41) and wild boars (*n* = 17) (Figure 1, right panel). In CdO, the maximum number of samples was collected in 2014 (*n* = 51), the minimal number in 2009 (*n* = 11) with no clear trend, whereas in NAq, the number of selected samples varied between 8 in 2010 and 69 in 2017 with a trend toward an increase with time (Figure 1).

### 2.3 Sequencing and alignment

Thermolysates of selected isolates were sequenced by Illumina sequencing (paired‐end 2*250 bp) at Genoscreen (Pasteur Institute, Lille) for CdO and Illumina sequencing (paired‐end 2*150 bp) at the Paris Brain Institute (ICM) for NAq. Sequencing quality was controlled using FASTQC with an acceptability Phred score threshold of 30. Sequence alignment and Single‐nucleotide polymorphism (SNP) calling were computed at the NRL using the Mb3601 reference strain (Branger et al., 2020) on Bionumerics software, version 7.6 (AppliedMath, Belgium). Identified SNPs were selected according to strict criteria of the wgSNP module: (1) they had to be present on at least five reads in both forward and reverse direction, (2) 12 base pairs had to separate them, (3) they were not present in repetitive regions of the genome, and (4) ambiguous SNPs (at least one unreliable (N) base, ambiguous (non ATCG) base or gap) were not included. SNPs were then used to reconstruct a maximum parsimony tree on Bionumerics to identify genetic outliers.

### 2.4 Bayesian phylogenetic modeling

We used BEAST2 (Bayesian evolutionary analysis by sampling trees) 2.6.4 to model bTB evolutionary (Bouckaert et al., 2019). The sequences for each region were analyzed separately.

### 2.5 The structured coalescent model

The differences in surveillance protocols between cattle and wildlife induce sampling biases. Indeed, wildlife cannot be exhaustively monitored, while all slaughtered cattle are tested for bTB. To take into account this sampling bias, we used the approximation of the structured coalescent as implemented in the Mascot (Marginal Approximation of the Structured COalescenT) 2.1.2 package (Müller et al., 2018). Indeed structured coalescent population models, contrary to migration models, are less susceptible to sampling bias (Müller et al., 2017). We assumed constant between‐species transition rates in times and used the Bayesian stochastic search variable selection (BSSVS) procedure to select only transition rates that explain the transition of *M. bovis* between the different species (Lemey et al., 2009). This procedure was designed to limit the number of transition rates to infer only those adequately explaining the diffusion between the subpopulations (here the host species) (Lemey et al., 2009). The estimated transition rates were backward in time, however, to avoid confusion we present the transition rates as the forward transition rates thereafter.

### 2.6 Substitution and molecular clock model selection

To select the best‐fitting model, the marginal likelihood (ML) was computed using the nested sampling algorithm as implemented in the NS 1.1.0 package (Russel et al., 2019). Models were then compared two by two by computing the Bayes factor (BF) as BF = log(ML2)‐log(ML1), where ML1 and ML2 are the ML of models 1 and 2, respectively. The level of support was considered overwhelming when |BF | > 150, strong if 20 < |BF | ≤ 150, positive 3 < |BF | ≤ 20 and hardly worth mentioning if 1≤|BF | ≤ 3 (Kass & Raftery, 1995). The model with the largest ML was favored. We tested the substitution models (JC69, HKY, and GTR) and three molecular clock models (strict, uncorrelated exponential, and uncorrelated lognormal relaxed molecular clock).

### 2.7 Parameters inference

To infer the parameters, we set the Markov chain Monte Carlo (MCMC) length to 50 million, the burn‐in to 10%, and sampled every 5000 iterations. Four replicates were performed. For each replicate, we inspected each parameter trace, looking for a stationary distribution, as well as the effective sample size (ESS). We considered ESS > 200 would guarantee that samples were independent and only selected models for each of the inferred parameters. We combined the four replicates with LogCombiner v2.6.6 with a lower sampling frequency to analyze thereafter 10,000 parameters. All trees were plotted using the ggtree package in R (Yu et al., 2017).

### 2.8 Host species transition

To study host species transitions, we resampled 1001 trees. We recorded for each node of each tree the predicted host species, its probability, and its height. We considered that the host species was known if its probability was >0.9 and unknown otherwise. We then computed the number of host species transitions between two consecutive nodes as well as the number of host species persistence (i.e., when the descendant host species is the same as the parental host species). If the host species for the parent and/or the descendant node was unknown, then the transition was considered unknown as well. We computed the host species transition and persistence at the lineage level. It is noteworthy that two consecutive nodes do not necessarily represent two distinct hosts; indeed these two nodes could represent strains hosted by the same individual or conversely strains that could have been transmitted to one or several other individuals. We computed the average number of transitions per tree for each parental node time as the sum of each transition or persistence divided by the total number of trees.

## 3 RESULTS

From the 144 SB0120‐CdO strains selected in CdO and from the 219 SB0120‐NAq strains selected in NAq, 123 and 290 SNPs were identified relative to the Mb3601 reference strain, respectively. When multiple isolates were available for a given outbreak, two or three unique SNPs sequences were detected in 39.7% of CdO outbreaks and 51.7% of NAq outbreaks.

### 3.1 Bayesian phylogenetic estimates

**Figure 2:**
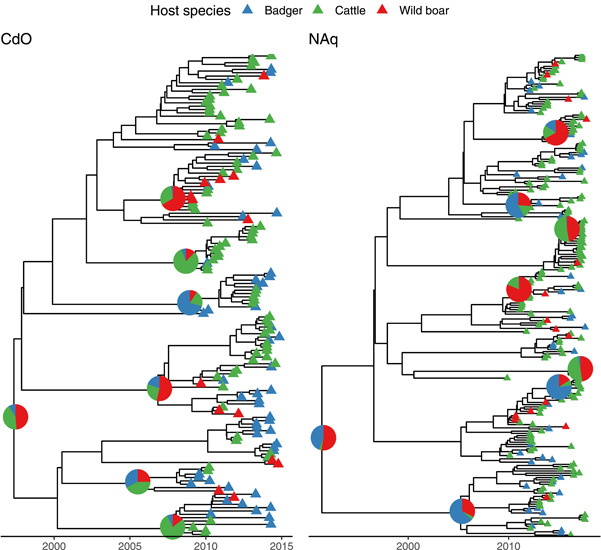
Phylogenetic trees for Côte‐d'Or (left) and Nouvelle‐Aquitaine (right). The tips are shown by triangles whose colors represent the species. The pies represent the median probability that the internal nodes were hosted by the three different species (badger in blue, cattle in green and wild boar in red). Pies were drawn for nodes with posterior probabilities >0.9.

According to the parameters, trace inspection showing stationary distribution, and ESS < 200, all models fitted the data well. The best‐fitted model, was an HKY substitution model, with an uncorrelated lognormal molecular clock for both study areas (Table A2). The corresponding inferred maximum clade credibility trees are shown in Figure 2.

According to the best‐fitting model for CdO, the most recent common ancestor (MRCA) of the studied isolates circulated in 1997 (with a 95% credibility interval between 1989 and 2002), as suggested by the median tree height (17.5 years, 95% highest posterior density (HPD): [12.6, 25.1]). It is however unclear whether the MRCA was hosted by wildlife or cattle (median host probability: 0.49 (95% HPD = [0, 0.97]) for wild boar, 0.35 (95% HPD = [0, 0.97]) for cattle, and 0.05 (95% HPD = [0, 0.33]) for badger). In NAq, the best‐fitting model predicted that the MRCA of the studied isolates circulated in 1991 (with a 95% credibility interval between 1975 and 1999), according to the median tree height (25.4 years, 95% HPD: [18.1, 42.0]). While the HPD intervals are overlapping, the predicted MRCA would have been hosted in wildlife (host probability: 0.55 (95% HPD = [0.02; 0.94]) for wild boar, *vs*. 0.40 (95% HPD = [0.01; 0.92]) for badger and 0.02 (95% HPD = [0; 0.11]) for cattle). The mean molecular clock was significantly (*p* < 0.001, Wilcoxon test) smaller in CdO with a median estimate of 0.42 substitution/genome/year (95%HPD: [0.31, 0.54]) versus 0.57 substitution/genome/year (95% HPD: [0.44, 0.71]) for NAq.

For both study areas, we inferred that the effective population size was the largest for badgers (CdO: median of 9.4, 95% HPD: [4.9, 14.6]; NAq: median of 21.2, 95% HPD: [13.4, 29.6]) before cattle (CdO: median of 3.8, 95% HPD: [1.6, 6.6]; NAq: median of 5.0, 95%HPD: [1.8, 9.6]) and wild boars (CdO: median of 2.7, 95% HPD: [0.3, 7.0]; Naq: median of 2.0, 95% HPD: [0.3, 5.3]). This means that two lineages hosted by badgers are expected to coalesce more slowly than two lineages hosted by cattle or wild boar because the coalescent rate is the inverse of the effective population size.

### 3.2 Host species transition

Table 1 shows the proportion of transition rates selected (i.e., different from zero) by the BSSVS procedure as well as the inferred transition rates median and HPDs when these rates were selected. We defined the selection threshold as the proportion of transition rates selected by the BSSVS procedure. For example, a selection threshold of 0.5 for a given transition rate means that this transition rate was selected by the BSSVS procedure in half of the outputs. Considering an arbitrary selection threshold of 0.80, three transition rates were selected in CdO, namely from cattle to badgers, from badgers to wild boars, and from wild boars to cattle, and four in NAq which were the same as in CdO with the additional transition rate from badgers to cattle.

**Table 1:** Median transition rates for both study areas, Côte‐d'Or (CdO) and Dordogne/Haute‐Vienne (NAq)

Transition rate	CdO			NAq		
	Selected	Median	HPD	Selected	Median	HPD
From cattle to badgers	**0.99**	**0.27**	**0.08–0.54**	**0.80**	**0.17**	**0.03–0.36**
From wild boars to badgers	0.58	0.09	0.01–0.47	0.41	0.11	0.01–0.30
From badgers to cattle	0.63	0.09	0.01–0.40	**0.88**	**0.56**	**0.11–1.32**
From wild boars to cattle	**0.94**	**0.24**	**0.03–0.84**	**0.99**	**1.03**	**0.31–2.57**
From badgers to wild boars	**0.97**	**0.24**	**0.03–0.83**	**0.90**	**0.42**	**0.08–1.16**
From cattle to wild boars	0.60	0.13	0.01–0.62	0.42	0.25	0.01–1.07

*Note*: “Selected” represents the proportion transition rate selected by the BSSVS procedure (i.e., non‐zero). The median and HPD were computed for the selected transition rates.

Abbreviation: HPD, 95% highest probability distribution.

In both study areas, the inferred transition rates showed large variations. It is therefore difficult to conclude the frequency of each transition event. Globally, these results suggest that *M. bovis* migrated from cattle to badgers, from badgers to wild boars, and from wild boars to cattle. In addition, in NAq a transition back from badgers to cattle was also predicted.

**Figure 3:**
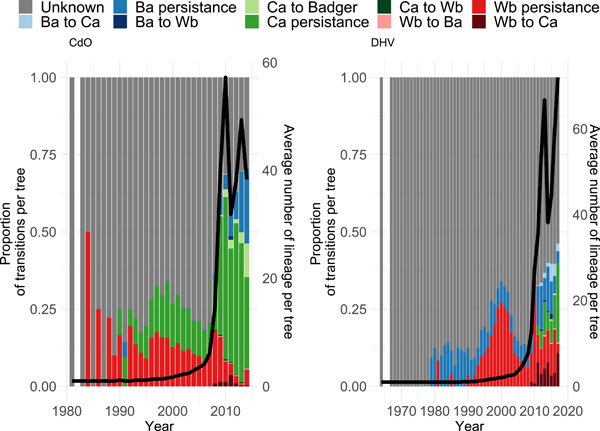
The proportion of transitions per tree and per year for CdO (left) and NAq (right). Computed from 1001 sampled posterior trees. Ca stands for Cattle, Ba for Badger, and Wb for Wild boar. Unknown transitions are considered when the posterior probability of the parent or descendant node is <0.9. The black line represents the average number of lineages per tree (right axis).

The proportion of unknown events, when the host species is considered unknown for a probability <0.9, is more important in NAq (0.89) than in CdO (0.36). For the known events, the majority is species persistence for both study areas with 0.90 in CdO (with 89.2% of persistence events being cattle persistence, 8.4% being badger persistence and 2.4% being wild boar persistence) and 0.88 in NAq (with 50.7% of persistence events being cattle persistence, 19.3% being badger persistence and 30.0% being wild boar persistence). Consistently with the inferred transition rate, the most frequent between‐species transition was cattle‐to‐badger which represented 95.2% of all between‐species transition events in CdO, and wild boar‐to‐cattle which represented 83.4% of all between‐species transition events in NAq. These results suggest that between‐species transitions remain rare events. Figure 3 shows the evolution with time of the average number of lineages and the proportion of transitions and persistence events per tree for both study areas. Moreover, it is worth mentioning that persistence events changed over time. Indeed, for both study areas, cattle persistence was most frequent and while badger persistence is identified onward starting as early as 1979 in NAq, it is gaining importance starting in 2008 only in CdO. On the opposite, cattle persistence is identified first in 1990 in CdO while only in 2009 in NAq.

## 4 DISCUSSION

In this study, we studied in two distinct study areas the transition of two different *M. bovis* strains belonging to the Eu3 clonal group among three host species, namely cattle, badgers, and wild boars. For this purpose, we used a structured coalescent model to infer transition rates between these three subpopulations. We showed that while the transition events remain rare events, our model predictions suggest that wild boars may be intermediaries for the transmission of *M. bovis* from badgers to cattle in both CdO and NAq.

We selected the same Bayesian evolutionary models for both study areas. More specifically, we selected an HKY substitution model and a lognormal molecular clock. The HKY substitution model has been previously used to model *M. bovis* phylogeny in cattle and brushtail possums in New Zealand (Crispell et al., 2017) and elks, white‐tailed deer, and cattle in Michigan, USA (Salvador et al., 2019). We estimated two significantly different substitution rates for both regions. Both estimates were higher than previously estimated in Northern Ireland from cattle and badgers (Biek et al., 2012; Trewby et al., 2016) and in Michigan from cattle and elk (Salvador et al., 2019). However, the substitution rates estimated from cattle and possums in New Zealand are in the same order of magnitude as the one we estimated from NAq (Crispell et al., 2017) and the one estimated from the South‐West of France from cattle and badgers (Duault et al., 2022) is in the same order of magnitude as the one we estimated for CdO. While, as noted by Duault et al., the differences between the studies could result from the different *M. bovis* lineage or the sampled species (Duault et al., 2022), this does not explain the significantly higher substitution rates inferred in NAq than in CdO. Indeed, for both study areas, the same host species were sampled during the same period. However, even if the spoligotype was the same in both study areas, the VNTR profiles differed. Hauer et al. showed that while SB0120 spoligotype was found all over France, specific VNTR profiles that spread locally were identified (such as 5 5 4 3 11 4 5 6 in CdO and 5 3 5 3 9 4 5 6 in NAq) (Hauer et al., 2015). While belonging to the Eu3 clonal group and sharing the same spoligotype, these strains were identified on different phylogenetic branches (Hauer et al., 2019) suggesting that VNTR profile and substitution rates could represent specific lineage characteristics.

To infer the internal nodes for host species, we used a structured coalescent method, which limits the impact of sampling bias (Müller et al., 2017). Indeed, contrary to the migration method (Lemey et al., 2009), structured coalescent methods do not assume that the migration process and tree‐generating process are independent. According to the selected transition rates between badgers and cattle, the relationship between both species was clearly different between the two study areas: transition rate from cattle to badgers was more frequently predicted than from badgers to cattle in CdO, contrary to NAq where both transition rates were predicted in more than 80% of the outputs. This could relate to the implementation of bTB biosecurity measures toward wildlife that were more stringent in CdO than in NAq. Recent studies including samples collected from two species, namely badger and cattle, are in favor of the transition from badger to cattle (Crispell et al., 2019; Duault et al., 2022; van Tonder et al., 2021), however in one of these studies, while the overall badger‐to‐cattle transition rate was higher than the cattle‐to‐badger transition rate, a more refined analysis on transmission cluster levels revealed that for 4/12 clusters, cattle‐to‐badger transition rates were higher than badger‐to‐cattle transition ones (van Tonder et al., 2021). Moreover, an epidemiological study reconstructing the contact network between badger setts and cattle farms concluded the intermediary role of badgers in *M. bovis* transmission in the South‐West of France (Bouchez‐Zacria et al., 2018). In addition, according to the French graphic parcel register (Relevé parcellaire Graphique, https://www.geoportail.gouv.fr), the landscape in NAq is more fragmented with numerous interfaces between pastures and wooded areas, than in CdO. Fragmented landscapes were shown to be associated with lower adult badger population densities, which was corroborated by their lower densities in NAq than in CdO (Jacquier et al., 2021). However, the increased interfaces between pastures and wooded areas could increase the contact rates between badgers and cattle in NAq (Bouchez‐Zacria et al., 2017) and explain the transition back and forth between these two species in this area compared to CdO. Also, bTB apparent prevalence in badgers might have increased between 2014 and 2016–2017 in NAq, while it has decreased in CdO during the same time interval (Réveillaud et al., 2018). The lack of evidence for the badger‐to‐cattle transition could also result from a lower statistical power due to the different sampling schemes in CdO and NAq.

It is noteworthy that we studied for the first time a multi‐host system including wild boars in addition to badgers and cattle. We showed that among the selected transition rate in both NAq and CdO, the transition rate from wild boar to cattle was identified, suggesting a relatively frequent transition from wild boar to cattle and the intermediary role played by wild boars between badgers and cattle. This highlight the important role played by wild boars in the spread of *M. bovis* to other species. This could be related to the large distances traveled by wild boars (Podgórski et al., 2013) compared to badgers (Byrne et al., 2014). In addition, cattle movement, even if less frequent, could also enhance *M. bovis* spread between distant farms (Palisson et al., 2016). Furthermore, in both study areas host species persistence was identified as the main event while between‐species transition represented less than 12% of known events, highlighting that between‐species transition remains a rare event.

To summarize, we showed that while wild boars played the role of intermediary host between badgers and cattle, the role of badgers differed between both regions: in CdO badgers were intermediaries from cattle to wild boars, whereas in NAq, badgers transmitted to both cattle and wild boars. Several factors could explain these differences. Some of them are inherent to our study design, such as the lineage that can lead to different substitution rates, the temporal depth, or the number of strains collected, while other relates to the landscape or the population density of wildlife species.

Our work has several limitations. First, we excluded red foxes, red deer, and roe deer samples from our study because of the limited number of available samples, which prevented us from evaluating their role in this multi‐host system. BTB‐infected foxes (*Vulpes vulpes*) were detected in Dordogne in 2015 (Michelet et al., 2018). A further study showed that in Dordogne, Landes, and Charente, bTB prevalence in foxes ranged between 5% and 10%, similar to that observed in badgers and wild boars (Richomme et al., 2020). However, due to the lack of information concerning red foxes, we cannot conclude about their role. Second, the sampling procedure varies between cattle and wildlife, with nearly exhaustive testing of cattle while samples collected from wildlife depend on events such as hunting or road kills leading to an underestimation of *M. bovis* infection prevalence in wildlife. In addition, wildlife carcasses are subject to contamination and deterioration lowering the culture sensitivity and thus poorer statistical representativeness in our sample compared to cattle (Rivière et al., 2015). While inference with structured coalescent models is less altered by sampling bias, unsampled demes (such as the red foxes or cervids in our study) could reshape our results. Thirdly, our approach did not allow the inclusion of spatialized information that could describe the localization of sampled isolates or the different sizes of the home range for badgers and wild boars, or the change of pastures for cattle. Finally, a large number of transition events was labeled as unknown. This is particularly true for NAq where nearly 90% of transition could not be labeled. The results concerning host species persistence and between‐species transition should therefore be considered with caution.

## 5 CONCLUSIONS

In conclusion, using a Bayesian evolutionary model, we inferred transition rates between cattle, badgers, and wild boars. Although this approach does not allow us to quantify within‐species transmission, our result shed light on the wild boar role, which appears to act as an intermediary between badgers and cattle in the circulation of two different Eu3 *M. bovis* in two distinct study areas.

## AUTHOR CONTRIBUTIONS

**Laetitia Canini**: Data curation (equal); formal analysis (equal); methodology (equal); validation (equal); visualization (equal); writing – original draft (equal). **Gabriela Modenesi**: Data curation (supporting); formal analysis (supporting); visualization (supporting); writing – original draft (supporting). **Aurélie Courcoul**: Conceptualization (equal); funding acquisition (equal); writing – original draft (equal). **Maria‐Laura Boschiroli**: Conceptualization (equal); project administration (equal); resources (equal); supervision (equal); writing – original draft (equal). **Benoit Durand**: Conceptualization (equal); funding acquisition (equal); supervision (equal); writing – original draft (equal). **Lorraine Michelet**: Conceptualization (equal); formal analysis (equal); funding acquisition (equal); investigation (equal); methodology (equal); project administration (equal); writing – original draft (equal).

## CONFLICT OF INTEREST

None declared.

## ETHICS STATEMENT

None required.

## Data Availability

All data are provided in full in the results section of this paper apart from all WGS data which are available in NCBI GenBank under BioProject PRJEB46102 for NAq: https://www.ncbi.nlm.nih.gov/bioproject/PRJEB46102 and PRJEB46417 for CdO: https://www.ncbi.nlm.nih.gov/bioproject/PRJEB46417. The individual isolates can be accessed under the following BioSample accession numbers: SAMEA8955321 ‐ SAMEA8955540 for NAq and SAMEA8987071 ‐ SAMEA8987214 for CdO.

## 1

**Table A1:** Accession numbers

DA	Sample	Collection date	Host scientific name	Biosample	Accession
10‐00086‐00	10Z000099	2009‐11‐09	*Bos taurus*	SAMEA8955321	ERR6198117
10‐00566‐00	10Z002121	2010‐02‐12	*Bos taurus*	SAMEA8955322	ERR6198204
10‐01026‐00	10Z005043	2010‐03‐26	*Bos taurus*	SAMEA8955323	ERR6198207
D‐10‐02190	10Z008544	2010‐09‐18	*Sus scrofa*	SAMEA8955324	ERR6198208
D‐10‐02191	10Z008545	2010‐09‐10	*Bos taurus*	SAMEA8955325	ERR6198209
D‐10‐02192	10Z008546	2010‐08‐15	*Sus scrofa*	SAMEA8955326	ERR6198210
D‐10‐02315	10Z009059	2010‐09‐12	*Sus scrofa*	SAMEA8955327	ERR6198216
D‐10‐02451	10Z009733	2010‐10‐18	*Meles meles*	SAMEA8955328	ERR6198228
D‐11‐00916	11Z001983	2011‐01‐04	*Bos taurus*	SAMEA8955329	ERR6198247
D‐11‐01150	11Z002149	2011‐02‐08	*Bos taurus*	SAMEA8955330	ERR6198291
D‐11‐01158	11Z002157	2011‐02‐27	*Meles meles*	SAMEA8955331	ERR6198317
D‐11‐01374	11Z002528	2011‐02‐22	*Bos taurus*	SAMEA8955332	ERR6198349
D‐11‐01375	11Z002529	2011‐03‐01	*Bos taurus*	SAMEA8955333	ERR6198365
D‐11‐01905	11Z003570	2011‐04‐12	*Bos taurus*	SAMEA8955334	ERR6198374
D‐11‐01992	11Z003734	2011‐05‐03	*Bos taurus*	SAMEA8955335	ERR6198375
D‐11‐02082	11Z003894	2011‐05‐05	*Bos taurus*	SAMEA8955336	ERR6198376
D‐11‐03278	11Z006170	2011‐10‐10	*Bos taurus*	SAMEA8955337	ERR6198377
D‐12‐00020	12Z000029	2011‐10‐10	*Meles meles*	SAMEA8955338	ERR6198378
D‐12‐01240	12Z002503	2012‐01‐11	*Meles meles*	SAMEA8955339	ERR6198379
D‐12‐01581	12Z003372	2012‐01‐17	*Bos taurus*	SAMEA8955340	ERR6198389
D‐12‐01582	12Z003373	2013‐01‐22	*Meles meles*	SAMEA8955341	ERR6198390
D‐12‐01589	12Z003430	2012‐03‐12	*Sus scrofa*	SAMEA8955342	ERR6198391
D‐12‐01597	12Z003438	2012‐01‐27	*Bos taurus*	SAMEA8955343	ERR6198393
D‐12‐01601	12Z003442	2012‐01‐24	*Bos taurus*	SAMEA8955344	ERR6198394
D‐12‐01697	12Z003519	2012‐02‐14	*Bos taurus*	SAMEA8955345	ERR6198395
D‐12‐01854	12Z004182	2012‐02‐13	*Meles meles*	SAMEA8955346	ERR6198396
D‐12‐01929	12Z004424	2012‐02‐14	*Bos taurus*	SAMEA8955347	ERR6198398
D‐12‐02001	12Z004494	2012‐03‐16	*Bos taurus*	SAMEA8955348	ERR6198399
D‐12‐02007	12Z004505	2012‐02‐09	*Bos taurus*	SAMEA8955349	ERR6198400
D‐12‐02120	12Z004741	2012‐03‐23	*Bos taurus*	SAMEA8955350	ERR6198401
D‐12‐02121	12Z004742	2012‐02‐28	*Bos taurus*	SAMEA8955351	ERR6198442
D‐12‐02129	12Z004749	2012‐03‐13	*Bos taurus*	SAMEA8955352	ERR6198443
D‐12‐02131	12Z004751	2012‐03‐13	*Bos taurus*	SAMEA8955353	ERR6198444
D‐12‐02240	12Z004928	2012‐03‐16	*Bos taurus*	SAMEA8955354	ERR6198445
D‐12‐02242	12Z004930	2012‐03‐27	*Bos taurus*	SAMEA8955355	ERR6198447
D‐12‐02292	12Z004995	2012‐03‐07	*Meles meles*	SAMEA8955356	ERR6198448
D‐12‐02293	12Z004996	2012‐03‐22	*Bos taurus*	SAMEA8955357	ERR6198449
D‐12‐02296	12Z004999	2012‐04‐02	*Meles meles*	SAMEA8955358	ERR6199027
D‐12‐02372	12Z005163	2012‐04‐06	*Meles meles*	SAMEA8955359	ERR6201794
D‐12‐02472	12Z005339	2012‐04‐05	*Meles meles*	SAMEA8955360	ERR6201795
D‐12‐02749	12Z005660	2012‐05‐05	*Meles meles*	SAMEA8955361	ERR6201796
D‐12‐02971	12Z006182	2012‐05‐22	*Bos taurus*	SAMEA8955362	ERR6201797
D‐12‐03180	12Z006994	2012‐05‐09	*Bos taurus*	SAMEA8955363	ERR6201798
D‐12‐03307	12Z007111	2013‐05‐24	*Meles meles*	SAMEA8955364	ERR6201800
D‐12‐03422	12Z007219	2012‐07‐23	*Bos taurus*	SAMEA8955365	ERR6201801
D‐12‐03423	12Z007220	2012‐07‐20	*Bos taurus*	SAMEA8955366	ERR6201802
D‐13‐00134	13Z000269	2012‐11‐16	*Meles meles*	SAMEA8955367	ERR6201804
D‐13‐00505	13Z001275	2013‐01‐03	*Meles meles*	SAMEA8955368	ERR6201806
D‐13‐00830	13Z001729	2013‐01‐25	*Bos taurus*	SAMEA8955369	ERR6201807
D‐13‐00831	13Z001730	2013‐01‐25	*Bos taurus*	SAMEA8955370	ERR6201808
D‐13‐00956	13Z001898	2013‐02‐01	*Bos taurus*	SAMEA8955371	ERR6201809
D‐13‐00962	13Z001903	2013‐02‐15	*Bos taurus*	SAMEA8955372	ERR6201810
D‐13‐01061	13Z001990	2013‐01‐02	*Bos taurus*	SAMEA8955373	ERR6201811
D‐13‐01238	13Z002208	2013‐02‐20	*Bos taurus*	SAMEA8955374	ERR6201812
D‐13‐01245	13Z002215	2013‐02‐15	*Bos taurus*	SAMEA8955375	ERR6201813
D‐13‐01303	13Z002277	2013‐03‐08	*Bos taurus*	SAMEA8955376	ERR6201814
D‐13‐01556	13Z002699	2013‐03‐29	*Bos taurus*	SAMEA8955377	ERR6201815
D‐13‐01562	13Z002705	2013‐04‐12	*Bos taurus*	SAMEA8955378	ERR6201817
D‐13‐01565	13Z002708	2013‐04‐22	*Bos taurus*	SAMEA8955379	ERR6201818
D‐13‐01566	13Z002709	2013‐04‐22	*Bos taurus*	SAMEA8955380	ERR6201819
D‐13‐02048	13Z003478	2013‐06‐05	*Bos taurus*	SAMEA8955381	ERR6201820
D‐13‐02100	13Z003643	2013‐04‐23	*Bos taurus*	SAMEA8955382	ERR6201821
D‐13‐02101	13Z003644	2013‐06‐07	*Bos taurus*	SAMEA8955383	ERR6201822
D‐13‐02225	13Z003769	2013‐03‐22	*Bos taurus*	SAMEA8955384	ERR6201823
D‐13‐02226	13Z003770	2013‐06‐13	*Bos taurus*	SAMEA8955385	ERR6201825
D‐13‐02368	13Z004012	2013‐06‐13	*Bos taurus*	SAMEA8955386	ERR6201826
D‐13‐02401	13Z004051	2013‐06‐13	*Bos taurus*	SAMEA8955387	ERR6201828
D‐13‐02436	13Z004129	2013‐04‐23	*Bos taurus*	SAMEA8955388	ERR6201829
D‐13‐02620	13Z004667	2013‐06‐13	*Bos taurus*	SAMEA8955389	ERR6201830
D‐13‐02704	13Z004785	2013‐08‐06	*Bos taurus*	SAMEA8955390	ERR6201831
D‐13‐03134	13Z005631	2013‐06‐13	*Bos taurus*	SAMEA8955391	ERR6201832
D‐13‐03137	13Z005634	2013‐08‐22	*Sus scrofa*	SAMEA8955392	ERR6201833
D‐13‐03140	13Z005637	2013‐09‐15	*Sus scrofa*	SAMEA8955393	ERR6201835
D‐13‐03227	13Z005787	2013‐09‐03	*Bos taurus*	SAMEA8955394	ERR6201836
D‐13‐03897	13Z010139	2013‐10‐29	*Bos taurus*	SAMEA8955395	ERR6201837
D‐13‐03898	13Z010140	2013‐11‐15	*Bos taurus*	SAMEA8955396	ERR6201838
D‐13‐03899	13Z010141	2013‐10‐13	*Sus scrofa*	SAMEA8955397	ERR6201839
D‐13‐03900	13Z010142	2013‐11‐10	*Sus scrofa*	SAMEA8955398	ERR6201840
D‐14‐00007	14Z000006	2013‐11‐03	*Sus scrofa*	SAMEA8955399	ERR6201841
D‐14‐00013	14Z000010	2013‐11‐20	*Bos taurus*	SAMEA8955400	ERR6201842
D‐14‐00016	14Z000013	2013‐11‐19	*Bos taurus*	SAMEA8955401	ERR6201843
D‐14‐00019	14Z000015	2013‐11‐19	*Bos taurus*	SAMEA8955402	ERR6201844
D‐14‐00020	14Z000016	2013‐11‐19	*Bos taurus*	SAMEA8955403	ERR6201845
D‐14‐01072	14Z003302	2013‐12‐22	*Meles meles*	SAMEA8955404	ERR6201847
D‐14‐01074	14Z003304	2014‐01‐13	*Meles meles*	SAMEA8955405	ERR6201848
D‐14‐01623	14Z004188	2014‐03‐28	*Bos taurus*	SAMEA8955406	ERR6201849
D‐14‐01757	14Z004539	2014‐03‐13	*Bos taurus*	SAMEA8955407	ERR6201850
D‐14‐01758	14Z004540	2014‐03‐12	*Bos taurus*	SAMEA8955408	ERR6201851
D‐14‐02086	14Z005338	2014‐04‐28	*Bos taurus*	SAMEA8955409	ERR6201852
D‐14‐02099	14Z005351	2014‐04‐23	*Bos taurus*	SAMEA8955410	ERR6201853
D‐14‐02112	14Z005364	2014‐04‐23	*Bos taurus*	SAMEA8955411	ERR6201854
D‐14‐02116	14Z005365	2014‐04‐09	*Bos taurus*	SAMEA8955412	ERR6201855
D‐14‐02259	14Z005576	2014‐05‐06	*Meles meles*	SAMEA8955413	ERR6201857
D‐14‐02574	14Z006089	2014‐05‐25	*Meles meles*	SAMEA8955414	ERR6201858
D‐14‐02811	14Z006436	2014‐06‐23	*Bos taurus*	SAMEA8955415	ERR6201860
D‐14‐02871	14Z006581	2014‐07‐24	*Bos taurus*	SAMEA8955416	ERR6201861
D‐14‐03006	14Z006798	2014‐06‐05	*Meles meles*	SAMEA8955417	ERR6201862
D‐14‐03118	14Z007047	2014‐06‐05	*Meles meles*	SAMEA8955419	ERR6201863
D‐14‐03380	14Z007947	2014‐09‐10	*Bos taurus*	SAMEA8955420	ERR6201864
D‐14‐03463	14Z008062	2014‐09‐10	*Bos taurus*	SAMEA8955421	ERR6201865
D‐14‐03644	14Z008376	2014‐09‐17	*Bos taurus*	SAMEA8955422	ERR6201866
D‐14‐03697	14Z008498	2014‐08‐24	*Sus scrofa*	SAMEA8955423	ERR6201868
D‐14‐04057	14Z009345	2014‐09‐21	*Sus scrofa*	SAMEA8955424	ERR6201869
D‐15‐00525	15Z001806	2015‐01‐14	*Bos taurus*	SAMEA8955425	ERR6201870
D‐15‐00613	15Z002145	2015‐01‐23	*Bos taurus*	SAMEA8955426	ERR6201871
D‐15‐00828	15Z002493	2015‐02‐13	*Bos taurus*	SAMEA8955427	ERR6201872
D‐15‐00970	15Z002980	2015‐02‐17	*Bos taurus*	SAMEA8955428	ERR6201873
D‐15‐01174	15Z004066	2015‐03‐06	*Bos taurus*	SAMEA8955429	ERR6201874
D‐15‐01176	15Z004068	2015‐03‐20	*Bos taurus*	SAMEA8955430	ERR6201875
D‐15‐01301	15Z004334	2015‐01‐25	*Meles meles*	SAMEA8955431	ERR6201877
D‐15‐01302	15Z004335	2015‐02‐27	*Bos taurus*	SAMEA8955432	ERR6201878
D‐15‐01311	15Z004344	2015‐03‐30	*Meles meles*	SAMEA8955433	ERR6201879
D‐15‐01312	15Z004345	2015‐04‐15	*Bos taurus*	SAMEA8955434	ERR6201881
D‐15‐01346	15Z004377	2015‐03‐12	*Meles meles*	SAMEA8955435	ERR6201882
D‐15‐01460	15Z004939	2015‐03‐13	*Meles meles*	SAMEA8955436	ERR6201883
D‐15‐01557	15Z005359	2015‐03‐22	*Meles meles*	SAMEA8955437	ERR6201884
D‐15‐01665	15Z005833	2015‐04‐17	*Bos taurus*	SAMEA8955438	ERR6201885
D‐15‐01667	15Z005835	2013‐05‐21	*Meles meles*	SAMEA8955439	ERR6201886
D‐15‐01759	15Z006243	2015‐05‐18	*Bos taurus*	SAMEA8955440	ERR6201887
D‐15‐01919	15Z006492	2015‐04‐08	*Meles meles*	SAMEA8955441	ERR6201888
D‐15‐01922	15Z006495	2015‐05‐18	*Bos taurus*	SAMEA8955442	ERR6201889
D‐15‐01960	15Z006541	2015‐05‐12	*Meles meles*	SAMEA8955443	ERR6201890
D‐15‐02070	15Z006760	2015‐06‐17	*Meles meles*	SAMEA8955444	ERR6201891
D‐15‐03139	15Z010210	2015‐10‐14	*Bos taurus*	SAMEA8955445	ERR6201893
D‐15‐03409	15Z010962	2015‐09‐13	*Sus scrofa*	SAMEA8955446	ERR6201894
D‐16‐00172	16Z000595	2015‐12‐15	*Bos taurus*	SAMEA8955447	ERR6201895
D‐16‐00301	16Z000964	2015‐12‐13	*Sus scrofa*	SAMEA8955448	ERR6201896
D‐16‐00302	16Z000965	2015‐12‐26	*Sus scrofa*	SAMEA8955449	ERR6201897
D‐16‐00361	16Z001097	2015‐12‐15	*Bos taurus*	SAMEA8955450	ERR6201898
D‐16‐01021	16Z002887	2016‐02‐15	*Bos taurus*	SAMEA8955451	ERR6201899
D‐16‐01467	16Z004107	2016‐03‐11	*Bos taurus*	SAMEA8955452	ERR6201901
D‐16‐01566	16Z004271	2016‐03‐25	*Bos taurus*	SAMEA8955453	ERR6201902
D‐16‐01567	16Z004272	2016‐03‐20	*Meles meles*	SAMEA8955454	ERR6201903
D‐16‐01569	16Z004274	2016‐03‐10	*Bos taurus*	SAMEA8955455	ERR6201906
D‐16‐01664	16Z004560	2016‐03‐22	*Bos taurus*	SAMEA8955456	ERR6201907
D‐16‐01669	16Z004566	2016‐03‐08	*Bos taurus*	SAMEA8955457	ERR6201908
D‐16‐01672	16Z004569	2016‐02‐17	*Meles meles*	SAMEA8955458	ERR6201909
D‐16‐01723	16Z004699	2016‐03‐08	*Meles meles*	SAMEA8955459	ERR6201910
D‐16‐01847	16Z005204	2016‐04‐12	*Bos taurus*	SAMEA8955460	ERR6201915
D‐16‐01848	16Z005206	2016‐04‐05	*Bos taurus*	SAMEA8955461	ERR6201937
D‐16‐01849	16Z005207	2016‐04‐06	*Bos taurus*	SAMEA8955462	ERR6201945
D‐16‐01851	16Z005209	2016‐03‐25	*Meles meles*	SAMEA8955463	ERR6201960
D‐16‐01852	16Z005210	2016‐04‐11	*Bos taurus*	SAMEA8955464	ERR6201972
D‐16‐02216	16Z006257	2017‐03‐27	*Meles meles*	SAMEA8955465	ERR6201978
D‐16‐02405	16Z006707	2016‐05‐27	*Bos taurus*	SAMEA8955466	ERR6201986
D‐16‐02601	16Z007121	2016‐05‐10	*Meles meles*	SAMEA8955467	ERR6201998
D‐16‐02833	16Z007476	2016‐06‐20	*Bos taurus*	SAMEA8955468	ERR6202004
D‐16‐02834	16Z007477	2016‐06‐24	*Bos taurus*	SAMEA8955469	ERR6202040
D‐16‐02835	16Z007478	2016‐06‐27	*Bos taurus*	SAMEA8955470	ERR6202401
D‐16‐02836	16Z007479	2016‐07‐01	*Bos taurus*	SAMEA8955471	ERR6203392
D‐16‐02924	16Z007681	2016‐06‐20	*Bos taurus*	SAMEA8955472	ERR6204677
D‐16‐03225	16Z008197	2016‐07‐21	*Bos taurus*	SAMEA8955473	ERR6206178
D‐16‐03320	16Z008443	2016‐07‐17	*Meles meles*	SAMEA8955474	ERR6208726
D‐17‐00049	17Z000058	2016‐12‐01	*Bos taurus*	SAMEA8955475	ERR6208976
D‐17‐00050	17Z000059	2016‐12‐01	*Bos taurus*	SAMEA8955476	ERR6209044
D‐17‐00051	17Z000060	2016‐12‐01	*Bos taurus*	SAMEA8955477	ERR6209102
D‐17‐00055	17Z000064	2016‐11‐01	*Sus scrofa*	SAMEA8955478	ERR6209192
D‐17‐00269	17Z000349	2016‐12‐06	*Bos taurus*	SAMEA8955479	ERR6209278
D‐17‐00270	17Z000350	2016‐12‐06	*Bos taurus*	SAMEA8955480	ERR6209301
D‐17‐00270	17Z000351	2016‐12‐06	*Bos taurus*	SAMEA8955481	ERR6209309
D‐17‐00540	17Z000898	2016‐12‐01	*Bos taurus*	SAMEA8955482	ERR6209323
D‐17‐00563	17Z000914	2017‐01‐09	*Bos taurus*	SAMEA8955483	ERR6209335
D‐17‐00641	17Z001046	2017‐01‐20	*Bos taurus*	SAMEA8955484	ERR6209343
D‐17‐00710	17Z001177	2017‐01‐09	*Bos taurus*	SAMEA8955485	ERR6209344
D‐17‐00849	17Z001576	2017‐02‐03	*Bos taurus*	SAMEA8955486	ERR6209345
D‐17‐00850	17Z001577	2017‐02‐03	*Bos taurus*	SAMEA8955487	ERR6209346
D‐17‐00851	17Z001578	2017‐02‐02	*Bos taurus*	SAMEA8955488	ERR6209347
D‐17‐00854	17Z001581	2017‐02‐02	*Bos taurus*	SAMEA8955489	ERR6209348
D‐17‐01066	17Z002146	2017‐02‐07	*Bos taurus*	SAMEA8955490	ERR6209349
D‐17‐01071	17Z002151	2017‐02‐10	*Bos taurus*	SAMEA8955491	ERR6209350
D‐17‐01146	17Z002194	2017‐02‐15	*Bos taurus*	SAMEA8955492	ERR6209351
D‐17‐01148	17Z002196	2017‐02‐09	*Bos taurus*	SAMEA8955493	ERR6209352
D‐17‐01149	17Z002197	2017‐02‐10	*Bos taurus*	SAMEA8955494	ERR6209353
D‐17‐01150	17Z002198	2017‐02‐07	*Bos taurus*	SAMEA8955495	ERR6209422
D‐17‐01286	17Z002553	2017‐01‐18	*Bos taurus*	SAMEA8955496	ERR6209423
D‐17‐01306	17Z002558	2017‐02‐27	*Bos taurus*	SAMEA8955497	ERR6209424
D‐17‐01412	17Z002740	2017‐02‐07	*Bos taurus*	SAMEA8955498	ERR6209443
D‐17‐01416	17Z002744	2017‐03‐08	*Bos taurus*	SAMEA8955499	ERR6209444
D‐17‐01517	17Z002865	2017‐03‐23	*Bos taurus*	SAMEA8955500	ERR6209445
D‐17‐01610	17Z003109	2017‐03‐01	*Bos taurus*	SAMEA8955501	ERR6209491
D‐17‐01611	17Z003110	2017‐02‐10	*Bos taurus*	SAMEA8955502	ERR6209492
D‐17‐01695	17Z003235	2017‐04‐05	*Bos taurus*	SAMEA8955503	ERR6209493
D‐17‐01696	17Z003236	2017‐03‐28	*Bos taurus*	SAMEA8955504	ERR6209524
D‐17‐01698	17Z003238	2017‐03‐21	*Bos taurus*	SAMEA8955505	ERR6209526
D‐17‐01705	17Z003245	2017‐03‐27	*Bos taurus*	SAMEA8955506	ERR6209527
D‐17‐01929	17Z003568	2017‐03‐30	*Bos taurus*	SAMEA8955507	ERR6209542
D‐17‐01931	17Z003570	2017‐04‐13	*Bos taurus*	SAMEA8955508	ERR6209573
D‐17‐02078	17Z004077	2017‐04‐21	*Bos taurus*	SAMEA8955509	ERR6209576
D‐17‐02080	17Z004080	2017‐04‐20	*Bos taurus*	SAMEA8955510	ERR6210127
D‐17‐02089	17Z004088	2017‐04‐20	*Bos taurus*	SAMEA8955511	ERR6210128
D‐17‐02090	17Z004128	2017‐04‐19	*Bos taurus*	SAMEA8955512	ERR6210129
D‐17‐02088	17Z004275	2017‐04‐20	*Bos taurus*	SAMEA8955513	ERR6210130
D‐17‐02260	17Z004423	2017‐03‐29	*Bos taurus*	SAMEA8955514	ERR6210147
D‐17‐02262	17Z004425	2017‐04‐19	*Bos taurus*	SAMEA8955515	ERR6210148
D‐17‐02264	17Z004428	2017‐05‐02	*Bos taurus*	SAMEA8955516	ERR6210149
D‐17‐02335	17Z004525	2017‐04‐24	*Bos taurus*	SAMEA8955517	ERR6210150
D‐17‐02438	17Z005047	2017‐04‐26	*Bos taurus*	SAMEA8955518	ERR6210158
D‐17‐02580	17Z005388	2017‐04‐19	*Bos taurus*	SAMEA8955519	ERR6210161
D‐17‐02586	17Z005394	2017‐05‐09	*Bos taurus*	SAMEA8955520	ERR6210169
D‐17‐02587	17Z005395	2017‐05‐18	*Bos taurus*	SAMEA8955521	ERR6210175
D‐17‐02698	17Z005495	2017‐05‐22	*Bos taurus*	SAMEA8955522	ERR6210188
D‐17‐02699	17Z005496	2017‐05‐22	*Bos taurus*	SAMEA8955523	ERR6210195
D‐17‐02775	17Z005671	2017‐05‐30	*Bos taurus*	SAMEA8955524	ERR6210201
D‐17‐02902	17Z005849	2017‐06‐23	*Bos taurus*	SAMEA8955525	ERR6210207
D‐17‐03220	17Z006451	2017‐03‐13	*Meles meles*	SAMEA8955526	ERR6210212
D‐17‐03217	17Z006454	2017‐06‐12	*Bos taurus*	SAMEA8955527	ERR6210216
D‐17‐03251	17Z006498	2017‐07‐17	*Bos taurus*	SAMEA8955528	ERR6210223
D‐17‐03430	17Z006770	2017‐07‐17	*Bos taurus*	SAMEA8955529	ERR6210230
D‐17‐03431	17Z006771	2017‐07‐15	*Meles meles*	SAMEA8955530	ERR6210236
D‐17‐03656	17Z007283	2017‐08‐06	*Bos taurus*	SAMEA8955531	ERR6210252
D‐17‐03910	17Z007631	2017‐08‐24	*Bos taurus*	SAMEA8955532	ERR6210258
D‐17‐03912	17Z007633	2017‐08‐10	*Bos taurus*	SAMEA8955533	ERR6210259
D‐17‐03913	17Z007634	2017‐08‐10	*Bos taurus*	SAMEA8955534	ERR6210266
D‐17‐04408	17Z008463	2017‐08‐24	*Bos taurus*	SAMEA8955535	ERR6210267
D‐17‐04412	17Z008467	2017‐07‐09	*Meles meles*	SAMEA8955536	ERR6210268
D‐17‐04421	17Z008476	2017‐08‐14	*Meles meles*	SAMEA8955537	ERR6210269
D‐17‐04423	17Z008478	2017‐04‐27	*Meles meles*	SAMEA8955538	ERR6210270
D‐17‐04936	17Z010330	2017‐10‐01	*Sus scrofa*	SAMEA8955539	ERR6210271
D‐17‐05074	17Z010552	2017‐10‐29	*Sus scrofa*	SAMEA8955540	ERR6210272

**Table A2:** Model selection

Model	Substitution model	Molecular clock model	Marginal likelihood	SD	BF	$2\sqrt{{\mathrm{SD}}_{2}^{2}+{\mathrm{SD}}_{1}^{2}}$	Reference model
**CdO**							
M1CdO	JC69	Strict	−24554	0.09	‐	‐	‐
M2CdO	HKY	Strict	−24225	1.00	329	2.00	M1CdO
M3CdO	GTR	Strict	Does not converge				
M4CdO	HKY	Uncorrelated lognormal	−24222	0.84	3	2.61	M2CdO
M5CdO	HKY	Uncorrelated exponential	−24223	0.91	−1	2.48	M4CdO
**NAq**							
M1NAq	JC69	Strict	−87642	0.18	‐	‐	‐
M2NAq	HKY	Strict	−3400	9.41	84242	18.82	M1NAq
M3NAq	GTR	Strict	Does not converge				
M4NAq	HKY	Uncorrelated lognormal	−3350	8.87	50	25.86	M2NAq
M5NAq	HKY	Uncorrelated exponential	−87616	1.04	−84266	17.9	M4NAq

The marginal likelihood was computed with the Nested Sampling algorithm implemented in the BEAST NS package, with 10 particles. For all models, an unstructured coalescent population model was used. BF stands for Bayes Factor and was computed as ML1‐ML2 if ML1 is the marginal likelihood of model 1 and ML2 marginal likelihood of model 2. To distinguish between both MLs, the difference should be greater than $2\sqrt{{\mathrm{SD}}_{2}^{2}-{\mathrm{SD}}_{1}^{2}}$, where SD1 stands for the standard deviation of model 1 and SD2 standard deviation of model 2.

Abbreviations: BF, Bayes factor; ML1, marginal likelihood of Model 1; ML2, marginal likelihood of Model 2; SD1, standard deviation of Model 1; SD2, SD2 standard deviation of Model 2.
